# Can essential fatty acids reduce the burden of disease(s)?

**DOI:** 10.1186/1476-511X-7-9

**Published:** 2008-03-18

**Authors:** Undurti N Das

**Affiliations:** 1 UND Life Sciences, 13800 Fairhill Road, #321, Shaker Heights, OH 44120, USA; 2 ICICI Centre for Technologies in Public Health (ICTPH), ICICI Knowledge Park, ICICI Bank Tower, Level IV, 1-11-256, Street # 1, Begumpet, Hyderabad-500 016, India

## Abstract

Coronary heart disease, stroke, diabetes mellitus, hypertension, cancer, depression schizophrenia, Alzheimer's disease, and collagen vascular diseases are low-grade systemic inflammatory conditions that are a severe burden on health care resources. Essential fatty acids (EFAs) and their metabolites: eicosapentaenoic acid (EPA), docosahexaenoic acid (DHA), gamma-linolenic acid (GLA), dihomo-gamma-linolenic acid (DGLA), and arachidonic acid (AA) and their products: prostaglandin E_1_, prostacyclin, lipoxins, resolvins, and protectins suppress inflammation, augment healing, and are of benefit in the prevention and management of these conditions. Hence, supplementation of EFAs could reduce burden of these disease(s).

## Introduction

Coronary heart disease (CHD), stroke, diabetes mellitus, hypertension, cancer, depression, schizophrenia, Alzheimer's disease, and collagen vascular diseases are a severe burden on the health care system throughout the world [1]. The major determinants of these chronic diseases are tobacco smoking, inadequate physical activity, unhealthy diets, overweight/obesity, suboptimal levels of blood pressure, cholesterol, and plasma glucose [2-4]. It was estimated that a multidrug regimen comprising of a statin, aspirin, and two blood-pressure lowering medicines reduces about 17.9 million deaths from cardiovascular diseases [4]. But, any strategy that prevents stroke, cancer and other chronic diseases in addition to cardiovascular diseases is expected to reduce the burden of chronic diseases substantially. Low-grade systemic inflammation is one of the characteristic features of CHD, stroke, diabetes mellitus, hypertension, cancer, depression schizophrenia, Alzheimer's disease, and collagen vascular diseases implying that prevention or suppression of inflammation reduces burden of these diseases.

## Low-grade systemic inflammation in chronic diseases

Plasma C-reactive protein (CRP), tumor necrosis factor-α (TNF-α), and interleukin-6 (IL-6), markers of inflammation, levels are elevated in subjects with obesity, insulin resistance, essential hypertension, type 2 diabetes, and CHD [5-10], suggesting that low-grade systemic inflammation occurs in them. Alzheimer's disease, depression, and schizophrenia are also low-grade systemic inflammatory conditions since, elevated levels of pro-inflammatory cytokines occurs in the plasma and brains of these patients [11,12].

Chronic inflammation is a major causative factor of human malignancies. Pro-inflammatory cytokines influence tumor microenvironment and promote cell growth and survival and angiogenesis such that tumor cell growth is facilitated. Thus, immune system activation could be a double edged sword: immune surveillance may check tumor development whereas aberrant immune activation promotes malignant growth [13,14].

## Low-grade systemic inflammatory conditions are also essential fatty acid deficient states

**Figure 1:**
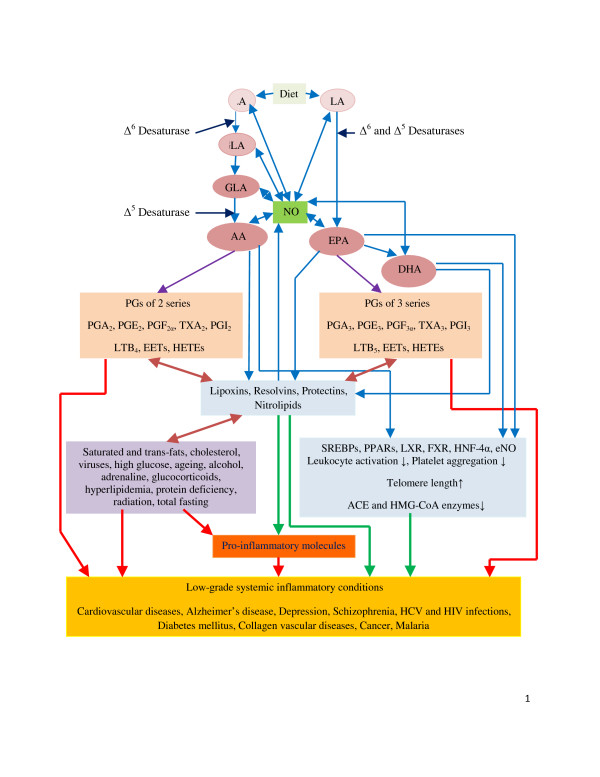
**Scheme showing metabolism of essential fatty acids and factors that modulate the formation of their metabolites and their actions.** Pro-inflammatory molecules include various PGs, LTs, TXs, and cytokines. For further details see text. NO = Endothelial Nitric oxide. Green line: Indicates prevention or suppression of disease and augmentation of healing process and inhibition of pro-inflammatory events or production of pro-inflammatory molecules. Red line: Indicates initiation or progression of disease, inflammatory process or augmentation of production of pro-inflammatory molecules. Double arrow: Indicates interaction between these molecules or feedback regulation. Eicosanoids formed from EPA are generally less inflammatory in nature compared to those formed from AA. Eicosanoids includes PGs, LTs, TXs, EETs, and HETEs.

Essential fatty acids (EFAs): *cis*-linoleic acid (LA, 18:2, ω-6) and α-linolenic acid (ALA, 18:3, ω-3) are important constituents of all cell membranes and alter membrane fluidity and thus, determine and influence the behaviour of membrane-bound enzymes and receptors. EFAs are essential and as are not synthesized in the body; have to be obtained in diet [15,16]. LA is converted to γ-linolenic acid (GLA, 18:3) by the enzyme Δ^6 ^desaturase (d-6-d) and GLA is elongated to form dihomo-GLA (DGLA, 20:3), the precursor of the 1 series of prostaglandins (PGs). DGLA can also be converted to arachidonic acid (AA, 20:4, ω-6) by the enzyme Δ^5 ^desaturase (d-5-d). AA forms the precursor of 2 series of PGs, thromboxanes (TXs) and the 4 series of leukotrienes (LTs). ALA is converted to eicosapentaenoic acid (EPA, 20:5) by d-6-d and d-5-d. EPA forms the precursor of the 3 series of PGs, TXs and the 5 series of LTs and docosahexaenoic acid (DHA). LA, GLA, DGLA, AA, ALA, EPA and DHA are all PUFAs, but only LA and ALA are EFAs (see Figure 1 for metabolism of EFAs). Although the terms EFAs and PUFAs are used interchangeably for the sake of convenience it should be understood that all EFAs are PUFAs but all PUFAs are not EFAs.

Most of the PGs, TXs, and LTs have pro-inflammatory actions. AA, EPA and DHA also give rise to anti-inflammatory molecules: lipoxins (LXs), resolvins, and protectins (such as neuroprotectin D1). Thus, PUFAs form precursors to both pro- and anti-inflammatory molecules and the balance between these mutually antagonistic compounds could determine the final outcome of the disease process (see Figure 1). In addition, nitration of unsaturated fatty acids occurs leading to the formation of nitrolipids that stimulate smooth muscle relaxation, block platelet activation, inhibit human neutrophil functions and suppress inflammation [15,16]. These studies suggest that PUFAs have important actions not only by themselves but also by giving raise to various biologically active compounds.

EFAs/PUFAs play a significant role in collagen vascular diseases, hypertension, diabetes mellitus, metabolic syndrome X, psoriasis, Alzheimer's disease, schizophrenia, depression, CHD, atherosclerosis, and cancer [17-25]. In all these conditions, plasma and tissue levels of GLA, DGLA, AA, EPA, and DHA are significantly low compared to normal suggesting that EFA/PUFA deficiency either predisposes or initiates the onset of these diseases [5,7,10-12,15-25]. EFAs/PUFAs play a significant role in such diverse conditions due to their ability to modulate cell membrane fluidity, possess second messenger action, influence angiotensin converting and HMG-CoA reductase enzymes, serve as ligands for nuclear receptors PPARs (peroxisome proliferator-activated receptors), LXR (liver X receptor), FXR (Farnesol X receptor), HNF-4α (hepatocyte nuclear factor-4α), enhance endothelial nitric oxide (eNO) generation, suppress leukocyte activation and production of pro-inflammatory cytokines IL-6 and TNF-α, prevent platelet aggregation, and regulate telomere length (reviewed in [26]). In addition, PUFAs form precursors to potent anti-inflammatory molecules: lipoxins, resolvins, and protectins that are essential to suppress inflammatory process and enhance healing [15,16].

In this context, it is noteworthy that saturated fats, cholesterol, trans-fatty acids, alcohol, adrenaline, and glucocorticoids inhibit Δ^6 ^and Δ^5 ^desaturases; pyridoxine, zinc, nicotinic acid, and magnesium are co-factors for normal Δ^6 ^desaturase activity; whereas insulin activates Δ^6 ^desaturase; and diabetics, hypertensives, and those with hyperlipidemias have reduced Δ^6 ^and Δ^5 ^desaturase (Δ^6 ^> Δ^5^) activities [15,16]. The activity of Δ^6 ^desaturase falls with age. Oncogenic viruses including hepatitis C virus and radiation inhibit Δ^6 ^desaturase activity. Total fasting, protein deficiency, and a glucose-rich diet reduce, whereas fat-free diet and partial caloric restriction enhance Δ^6 ^desaturase activity. Activity of Δ^6 ^and Δ^5 ^desaturases are regulated by sterol regulatory element binding protein-1 (SREBP-1) and peroxisome proliferator-activated receptor-α (PPAR-α), two reciprocal transcription factors for fatty acid metabolism, and some of their (SREBP-1 and PPAR-α) lipogenic functions are brought about by their action on PUFAs [27]. Interference with the metabolism of EFAs by saturated fats, cholesterol and trans fats, glucose, insulin deficiency, viruses, alcohol, and ageing reduces the formation of GLA, DGLA, AA, EPA, and DHA and their beneficial metabolites prostacyclin (PGI_2_), PGI_3_, lipoxins, resolvins, and protectins that could account for the initiation and progression of atherosclerosis, persistence of inflammation, CHD and failure of the healing process [15,16,19,24].

## PUFAs have anti-bacterial, anti-viral, anti-fungal, anti-parasitic actions

LA, ALA, and AA have bacteriostatic effect on both gram-positive and gram-negative bacteria [28]. Both LA and AA can inactivate animal herpes, influenza, Sendai, and Sindbis virus within minutes of contact [29].

LA, AA, EPA, and DHA induced death of *Plasmodium *both *in vitro *and *in vivo *[30-32]. An analog of myristic acid (14:0) showed selective toxicity to African Trypanosomes [33]. Prostaglandin E_1 _(PGE_1_) and PGA, derived from DGLA, AA, and EPA inhibited viral replication [34,35]. These results suggest that PUFAs function as endogenous anti-bacterial, anti-parasitic, and anti-viral molecules [36,37]. PUFAs augment the anti-bacterial actions of synthetic antibiotics against drug-resistant bacteria [38]. There is evidence to suggest that PUFAs can inactivate HIV (human immunodeficiency virus), an enveloped virus, and thus, is of benefit in AIDS (acquired immunodeficiency syndrome) [39,40]. Patients with AIDS and intravenous drug abusers have low plasma phospholipid DGLA, AA and DHA concentrations that could favour the onset and development of AIDS [41,42]. Lymphocytes and macrophages contain significant amounts of PUFAs and release them on appropriate stimulation. PUFAs stimulate NADPH-dependent superoxide production by macrophages, neutrophils and lymphocytes that has bactericidal action [43].

AA, DHA, and EPA exert anti-HCV (hepatitis C virus) activities at physiologically relevant concentrations. Strong synergistic anti-HCV effect was observed when AA was combined with IFN-α (interferon-α) [44-46]. The anti-HCV action of PUFAs is due to the formation of significant amounts of lipid peroxides. Previously, I observed that PUFA-induced tumoricidal action is also due to increased formation of lipid peroxides and free radical generation [47]. Thus, PUFAs have anti-bacterial, anti-fungal, anti-viral, anti-parasitic and tumoricidal actions.

## Hypothesis

It is evident from the preceding discussion that EFAs/PUFAs are not only naturally occurring endogenous substances present in almost all tissues and essential components of all mammalian cells but also useful in the prevention and treatment of low-grade systemic inflammatory conditions: atherosclerosis, CHD, stroke, diabetes mellitus, hypertension, cancer, depression, schizophrenia, Alzheimer's disease, and collagen vascular diseases if they are present in adequate amounts in various target tissues [15-26]; and function as endogenous anti-biotic-like molecules [36,37,44]. EFAs/PUFAs have aspirin-like action, inhibit of HMG-CoA and ACE enzymes, and possess diuretic, anti-hypertensive, and β-blocker-like actions, which suggests that they (PUFAs) function as endogenous "polypill" and thus, prevent cardiovascular mortality and morbidity [48].

Dietary intake of PUFAs (especially ω-3) from infancy reduced the risk for type 1 diabetes [49] confirming previous animal studies that PUFAs prevent chemical-induced diabetes [22,23]. Increasing concentrations of ω-6 DGLA and AA in breast milk reduced the risk of mother-to-child transmission of HIV [50] suggesting that PUFAs have anti-HIV actions [39,40]. Since many low-grade systemic inflammatory conditions may have their origins in the perinatal period [18], I propose that PUFAs be given to pregnant women and lactating mothers and to children from infancy throughout life to reduce the burden of both infectious and non-infectious diseases.

This hypothesis can be tested by studying the effect of supplementation of ω-3 and ω-6 fatty acids (in different combinations and doses) in animal models of various human diseases. It is envisaged in this hypothesis that the plasma and tissue concentrations of various PUFAs and their beneficial metabolites such as PGI_2_, PGE_1_, lipoxins, resolvins, and protectins will be lower in various low-grade systemic inflammatory conditions compared to normal. This hypothesis implies that in subjects who have lower normal levels and those who are marginally deficient in PUFAs are more likely to develop HCV, HIV, malaria, and bacterial infections. If this hypothesis is true, it indicates that those who fail to produce adequate amounts of lipoxins, resolvins, and protectins are less likely to recover from these diseases in time. Since, various PUFAs can be obtained from diet or supplemented from external sources; it will be interesting to study the therapeutic benefits of various ω-3 and ω-6 fatty acids in the diseases that have been enumerated above. It is important to study which type (ω-3, ω-6 or both) of fatty acids and in what combination or ratio and which form (oral or parenteral) are most suited to suppress or give relief from various diseases. It is relevant to study whether suitable synthetic analogues of various PUFAs can be developed for their possible therapeutic use in various diseases. Since most of the low-grade systemic inflammatory conditions have their origin in the perinatal period and childhood, it is important that studies are performed wherein PUFAs are given from infancy and wherever possible to the mother during pregnancy and lactation till adult age to know whether such long (sometimes even lifelong) term supplementation is necessary to suppress or postpone the development of the disease or at least decrease the severity of the disease in the event it occurs. Since PUFAs are relatively safe even when taken for long periods of time, such intervention studies in humans are not difficult. Preliminary studies [49,50] are already in support of such an intervention in humans.
